# Imaging Habenula Volume in Schizophrenia and Bipolar Disorder

**DOI:** 10.3389/fpsyt.2018.00456

**Published:** 2018-09-24

**Authors:** Matthew Schafer, Joo-Won Kim, Joshmi Joseph, Junqian Xu, Sophia Frangou, Gaelle E. Doucet

**Affiliations:** ^1^Graduate School of Biomedical Sciences, Icahn School of Medicine at Mount Sinai, New York, NY, United States; ^2^Translational and Molecular Imaging Institute, Icahn School of Medicine at Mount Sinai, New York, NY, United States; ^3^Department of Radiology, Icahn School of Medicine at Mount Sinai, New York, NY, United States; ^4^Department of Neuroscience, Icahn School of Medicine at Mount Sinai, New York, NY, United States; ^5^Department of Psychiatry, Icahn School of Medicine at Mount Sinai, New York, NY, United States

**Keywords:** habenula, segmentation, schizophrenia, bipolar disorder, suicidality, structural MRI

## Abstract

The habenula (Hb), a bilateral nucleus located next to the dorsomedial thalamus, is of particular relevance to psychiatric disorders based on preclinical evidence linking the Hb to depressive and amotivational states. However, studies in clinical samples are scant because segmentation of the Hb in neuroimaging data is challenging due to its small size and low contrast from the surrounding tissues. Negative affective states dominate the clinical course of schizophrenia and bipolar disorder and represent a major cause of disability. Diagnosis-related alterations in the volume of Hb in these disorders have therefore been hypothesized but remain largely untested. To probe this question, we used a recently developed objective and reliable semi-automated Hb segmentation method based on myelin-sensitive magnetic resonance imaging (MRI) data. We ascertained case-control differences in Hb volume from high resolution structural MRI data obtained from patients with schizophrenia (*n* = 95), bipolar disorder (*n* = 44) and demographically matched healthy individuals (*n* = 52). Following strict quality control of the MRI data, the final sample comprised 68 patients with schizophrenia, 32 with bipolar disorder and 40 healthy individuals. Regardless of diagnosis, age, sex, and IQ were not correlated with Hb volume. This was also the case for age of illness onset and medication (i.e., antipsychotic dose and lithium-treatment status). Case-control differences in Hb volume did not reach statistical significance; their effect size (Cohen's *d*) was negligible on the left (schizophrenia: 0.14; bipolar disorder: −0.03) and small on the right (schizophrenia: 0.34; bipolar disorder: 0.26). Nevertheless, variability in the volume of the right Hb was associated with suicidality in the entire patient sample (ρ = 0.29, *p* = 0.004) as well as in each patient group (bipolar disorder: ρ = 0.34, *p* = 0.04; schizophrenia: ρ = 0.25, *p* = 0.04). These findings warrant replication in larger samples and longitudinal designs and encourage more comprehensive characterization of Hb connectivity and function in clinical populations.

## Introduction

Schizophrenia (SCZ) and bipolar disorder (BD) consistently rank among the leading causes of disability worldwide (1). These disorders show similarities across multiple levels, including shared genetic risk factors (2, 3), overlapping brain structural (4–7) and cognitive deficits (8, 9) and common abnormalities in reality testing and affect regulation (10, 11). Amongst these multiple components of impairment, there is an increasing emphasis on the role of negative affective states because they dominate the clinical picture of both disorders. It is estimated that about 50% of patients with SCZ experience depression (12) while depressive symptoms are more pervasive than manic/hypomanic or mixed symptoms in the natural history of BD (13, 14). A similar overlap is noted for amotivational states as patients with either SCZ or BD seem to report similar rates of anhedonia, avolition and asociality (15, 16). Both disorders are also associated with increased suicidality in recent onset and in chronic patients (17–20).

Using the Research Domain Criteria (RDoC) framework (21), current mechanistic models of psychopathology in SCZ and BD implicate abnormalities in the Positive (PVS) and Negative Valence (NVS) Systems. In the case of SCZ, erratic signaling in response to reward is thought to disrupt stimulus-outcome associations resulting in inappropriate value (or salience) attribution to stimuli and predictive cues; consequently, appropriate reward responses are blunted leading to negative symptoms while responses to irrelevant stimuli are enhanced, leading to psychotic symptoms (22–25). In the case of BD, the prevailing hypothesis is that responsivity to rewarding stimuli and cues is increased in mania and blunted during depressive episodes (25, 26). Despite this progress, there are significant gaps to our understanding of valence processing in SCZ and BD and particularly with regards to the contribution of the Negative Valence Systems.

Although the PVS and NVS are considered separately within the RDoC framework, both animal and human studies indicate that their corresponding circuits intersect in the anterior (ACC) and posterior cingulate cortex (PCC), regions of the lateral and medial prefrontal cortex (PFC), insula, amygdala/hippocampus complex (AMG/HIPP), striatum, thalamus, ventral tegmental area (VTA) and habenula (Hb) (27–29). Within this extensive network, the Hb has a unique role as it preferentially signals negative outcomes or cues (30). The Hb is a bilateral nucleus, with lateral (LHb) and medial (MHb) subdivisions, located next to the dorsomedial thalamus. One histological study showed that the lateral subdivision is larger on the left than the right while no left-right differences have been noted for the medial subdivision in postmortem human brain (31). Notably, the asymmetrical lateralization of the Hb seems phylogenetically conserved (32, 33). Preclinical studies also suggest that the LHb and MHb differ in their anatomical connectivity with the rest of the brain. The LHb is mainly connected with forebrain areas, hypothalamus, the globus pallidus and ventral tegmental area and the medial PFC and interacts with dopaminergic, serotonergic and noradrenergic systems (34). The MHb primarily receives inputs from the medial and lateral septal nuclei while its efferent connections are almost entirely directed to interpeduncular nucleus (IPN) and is characterized by the abundance of nicotinic acetylcholine receptors (nAChRs) (34). In humans, the functional connectivity of the Hb largely follows its anatomical connections, but also extends to other neocortical regions, such as ACC, PCC, and dorsal PFC, the AMG/HIPP and the ventral striatum (35–37). Increased activity in the LHb neurons is thought to elicit aversion by exciting GABAergic neurons in the rostromedial tegmental nucleus thus reducing activity in the VTA dopaminergic neurons that project to the medial PFC (38). Thus the LHb has been shown to be reliably inhibited by rewarding cues and outcomes while being activated by negative stimuli and predictive cues (39–42). The MHb has been implicated in mood regulation based on the depressive-like behaviors associated with targeted genetic lesions of this region in rodents (34, 43). Additionally, inactivation of cholinergic input from the MHb to the nucleus accumbens (NAc) via the IPN has been linked to fear (44) and anhedonia (43, 45).

Despite the potentially important role of the Hb for negative affect processing in BD and SCZ, research on its morphology and function is generally sparse. This is partly due to the multiple methodological challenges in imaging the Hb *in vivo* because this region is very small and difficult to delineate from the adjacent thalamus (46–48). A single post-mortem study of the Hb in patients with Major Depressive Disorder (MDD; *n* = 6), BD (*n* = 8) and SCZ (*n* = 17) found that volume reduction in the Hb was associated with affective disorders (49). This association was contradicted in subsequent *in-vivo* imaging studies that were unable to find case-control differences in the Hb volume in patients with MDD (50, 51) but reported a significant reduction in patients with SCZ (52). A further study suggested that Hb volume reductions may be restricted to unmedicated BD patients and to depressed women with MDD (53). Key methodological concerns about the inconsistencies in the Hb volumetric studies relate to the small number of patients involved (range 16–34) and the reliance on manual Hb segmentation.

The primary aim of the current study was to test for Hb volume reductions in patients with either BD or SCZ compared to healthy individuals using a recently developed objective semi-automated Hb segmentation method with reliable and reproducible boundary definitions (46, 47). A secondary goal was to explore the relationship between the Hb volume with the severity of blunted affect, depression, emotional withdrawal and suicidality in patients with SCZ or BD given the association of the Hb with negative affective states.

## Methods

### Participants

We recruited individuals with SCZ (*n* = 95), individuals with BD, type I (*n* = 44), and healthy individuals (HI; *n* = 52). Patients were recruited via clinician referrals from the psychiatric services of the Mount Sinai Health System, NY. Healthy individuals were recruited via advertisement in the local press. The eligibility criteria for all participants were (a) 18–45 years; (b) English fluency; (c) IQ > 70; (d) no history of head trauma or loss of consciousness; (e) no current or lifetime history of medical or neurological disorders; (f) no lifetime history of substance use disorder; (g) no MRI contra-indications (e.g., metal implants, claustrophobia). In addition, patients fulfilled diagnostic criteria of either BD, type I or SCZ based on the Diagnostic and Statistical Manual of Mental Disorders (DSM-5) (54) while healthy individuals were included if they had no lifetime personal history of mental disorders and no family history (up to second-degree relatives) of SCZ or BD.

The diagnostic status of all participants was determined using the research version of the Structured Clinical interview for DSM-5 (55) supplemented by information from medical records in the case of patients. In all participants, the presence and severity of psychopathology in the preceding 2-weeks were assessed immediately prior to the scan using the expanded 24-item Brief Psychotic Rating Scale (BPRS) (56) which allows decomposition of the clinical profile into four dimensions comprising positive symptoms, negative symptoms, depression/anxiety and disorganization/mania (57, 58). Master's-level research coordinators with at least 2 years of clinical experience conducted the assessments following standardized training to ensure inter-rater reliability of at least 0.90 for diagnostic and psychopathology ratings. An estimate of IQ was obtained from all participants using the Wechsler Abbreviated Scale of Intelligence, 2nd Edition (WASI-II) (59). Medication type and dose was recorded in all patients and the daily antipsychotic dose was converted to chlorpromazine equivalents (CPZE) (60). The study was approved by the Institutional Review Board of the Icahn School of Medicine at Mount Sinai (ISMMS). All participants provided written informed consent.

### Neuroimaging acquisition

Structural imaging data (T1-weighted and T2-weighted) were acquired at ISMMS on a 3T Skyra scanner (Siemens, Erlangen, Germany) with a 32-channel receiver coil. Anatomical acquisitions were identical for all participants. The T1-weighted (T1w), 3D magnetization-prepared rapid gradient-echo (MPRAGE) sequence was acquired with the following parameters: Field of view (FOV) = 256 × 256 × 179 mm^3^, matrix size: 320 × 320, 0.8 mm isotropic resolution, Time to Echo (TE)/Repetition time (TR) = 2.07/2,400 ms, inversion time (TI) = 1,000 ms, 8° flip-angle with binomial (1, −1) fat saturation, bandwidth 240 Hz/Pixel, echo spacing 7.6 ms, in-plane acceleration (GRAPPA; GeneRalized Autocalibrating Partial Parallel Acquisition) factor 2 and total acquisition time of ~7 min. The T2-weighted (T2w), 3D variable-flip angle turbo-spin-echo (SPACE) sequence was acquired with the following parameters: FOV: 256 × 256 × 179 mm^3^, matrix size: 320 × 320, 0.8 mm isotropic resolution, TE/TR = 565/3,200 ms, 120° flip-angle, bandwidth 680 Hz/Pixel, echo spacing 3.87 ms, in-plane acceleration GRAPPA factor 2, turbo factor 314, and total acquisition time of ~7 min.

### Habenula extraction

We applied the FreeSurfer processing of the Human Connectome Project Pipelines (61) on the T1w and T2w images, including gradient non-linearity distortion correction (62), anterior commissure (AC)-posterior (PC) commissure alignment and T2w-to-T1w registration, but excluded bias field correction which reduces Hb-thalamus contrast (46). Myelin-sensitive images were created using T1w-to-T2w ratios (63).

From the myelin-sensitive, T1w and T2w images, we generated both binary segmentation and probability map of the left and right Hb using the objective semi-automated human Hb segmentation scheme (www.github.com/junqianxulab/habenula_segmentation). Information regarding the development and validation of this approach has been described in previous publications (46, 47). Specifically, (i) we have corroborated the method used here against Hb boundary definitions derived from *in vivo* 7T myelin-sensitive images (T1w over T2w ratio images), 7T *ex vivo* proton density-weighted images and images from the Allen Brain Atlas (http://www.brain-map.org/); (ii) an expert neuroanatomist (Dr. Thomas Naidich) reviewed and approved the segmented Hb boundaries in previous methodological studies (46, 47); (iii) we have tested inter-site reliability by obtaining 3T scans from the same healthy individuals (*n* = 12) at 3 different sites; (iv) we have affirmed inter-site reliability by obtaining 3T scans from 27 healthy individuals scanned twice at the Icahn School of Medicine at Mount Sinai with an interval of 2 weeks; and (v) we have shown better reliability of our method compared to the geometric method (48). Briefly, this scheme consists of five steps: (i) Hb region-of-interest initialization, (ii) intensity thresholding, (iii) region growing segmentation, (iv) lateral and inferior geometric constraint, and (v) partial volume estimation (46). The only manual process was to select an initialization voxel within each of the left and right Hb in step (i). The output of this algorithm provides a probabilistic map of the right and left Hb and Hb volumes in mm^3^. The structural processing and Hb extraction steps were done blind to diagnosis.

### Quality control

The Hb was covered by 4 coronal slices and 5 axial slices on average across all participants. There was no difference in the number of slices covering the right and left habenula or between the diagnostic groups. All scans were reviewed by a clinical radiologist to exclude incidental pathological findings including pineal gland cysts. None of the participants was excluded on these grounds. To minimize biases in the Hb segmentation, we applied strict quality control criteria blind to diagnosis. Each structural scan was included only if passing a quality control process that involved manual viewing (done by coauthors JJ and GD) and rating of each scan on a 4-point scale (1 = poor and 4 = excellent). A scan with a score = 4 did not show any evidence of head motion, significant ringing or blurriness, a scan with a score = 3 showed minor ringing, a scan with a score = 2 showed posterior blurriness, and a scan with a score = 1 showed significant blurriness due to severe head motion and ringing. These ratings were based on the quality control criteria and protocols developed by the Human Connectome Project (https://www.humanconnectome.org/storage/app/media/documentation/s1200/HCP_S1200_Release_Appendix_IV.pdf).

**Figure 1:**
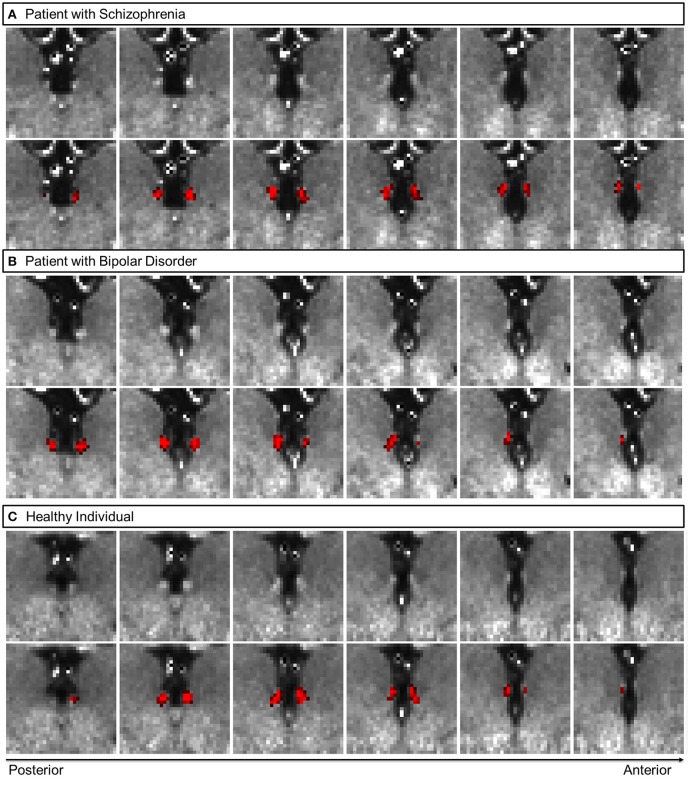
Representative coronal views of myelin-sensitive (T1w/T2w, top row) images, and the habenula segmentation probability map (bottom row, in red) for a patient with schizophrenia **(A)**, a patient with bipolar disorder **(B)** and a healthy participant **(C)**.

Participants with at least one poor quality scan (i.e., a rating of 1 or 2 for either T1w or T2w scans) were excluded from further analyses. After the Hb segmentation, the results were manually inspected (by coauthors JJ, JWK, and GD) for under- and over-estimation, and atypical shape (48). Following quality control, the final sample comprised 140 participants (73% from the initial sample; 68 patients with SCZ, 32 patients with bipolar disorder, 40 healthy participants) (Table 1). Excluded individuals were similarly distributed across diagnostic groups. The excluded participants did not differ compared to the remaining sample in terms of age (*p* = 0.2), sex (*p* = 0.8), total BPRS score (*p* = 0.3), blunted affect (*p* = 0.9), depression (*p* = 0.6), emotional withdrawal (*p* = 0.5) and suicidality (*p* = 0.9). Representative Hb segmentation probability maps for each diagnostic group are shown in Figure 1.

**Table 1:** Study sample characteristics.

**Variable**	**Healthy individuals *N* = 40**	**Patients with bipolar disorder *N* = 32**	**Patients with schizophrenia *N* = 68**
Age, mean (SD), years	29.78 (8.65)	28.19 (8.74)	27.64 (7.37)
Women, no (%)	16 (40%)	12 (37.5%)	19 (28%)
IQ, mean (SD)^a^	119.50 (15.65)	103.78 (16.91)	93.12 (16.0)
Age of onset, mean (SD), years	n/a	20.52 (4.75)	21.74 (5.03)
BPRS total score, mean (SD)^b^	24.16 (0.37)	46.83 (17.89)	50.94 (19.55)
BPRS positive symptoms score, mean (SD)^c^	4.0 (0)	9.90 (5.25)	12.87 (6.21)
BPRS negative symptoms score, mean (SD)^d^	3.03 (0.17)	4.21 (1.84)	6.67 (3.88)
BPRS depression/anxiety symptoms score, mean (SD)^b^	4.0 (0)	9.03 (4.42)	8.56 (4.97)
BPRS mania/disorganization symptoms score, mean (SD)^e^	5.0 (0)	11.37 (8.54)	8.00 (5.22)
Antipsychotic dose, mean (SD), CPZE^f^	n/a	232.48 (210.33)	269.86 (224.48)
Any antipsychotic, n (%)	n/a	27 (84%)	62 (91%)
First-generation antipsychotics, n (%)^*^^,^^*f*^	n/a	6 (19%)	12 (18%)
Second-generation antipsychotics, n (%)^*^^,^ ^*f*^	n/a	27 (84%)	56 (82%)
Antidepressants, n (%)^f^	n/a	8 (25%)	20 (29%)
Lithium, n (%)^g^	n/a	15 (47%)	4 (6%)
Anti-Epileptic n (%)^f^	n/a	6 (19%)	14 (21%)
Two or more medication classes, n (%)	n/a	19 (59%)	30 (44%)
No medication, n (%)	n/a	1 (3%)	2 (3%)

*BPRS, Brief Psychiatric Rating Scale; CPZE, chlorpromazine equivalents; n/a, not applicable*.

*In the BPRS symptoms are coded as 1 (absent) to 7 (extremely severe); BPRS Total Score, sum of scores of the 24 items; BPRS positive symptoms, sum of scores for hallucinations, unusual thought content, bizarre behavior items; BPRS negative symptoms, sum of scores for blunted affect, emotional withdrawal, motor retardation items; BPRS Depression/Anxiety Scores, sum of scores for anxiety, depression, suicidality, guilt items; BPRS Mania/Disorganization Scores, sum of scores for motor hyperactivity, elevated mood, excitement, distractibility, grandiosity items*.

* *Some patients were on both first and second-generation antipsychotics*.

*BD, Bipolar Disorder; HI, healthy individuals; SCZ, Schizophrenia*;

a *HI > BD > SCZ*;

b *SCZ = BD > HI*;

c *SCZ > BD > HI*;

d *SCZ > BD = HI*;

e *BD > SCZ > HI*;

f *BD = SCZ*;

g *BD > SCZ; based on appropriate tests at p < 0.05*.

**Figure 2:**
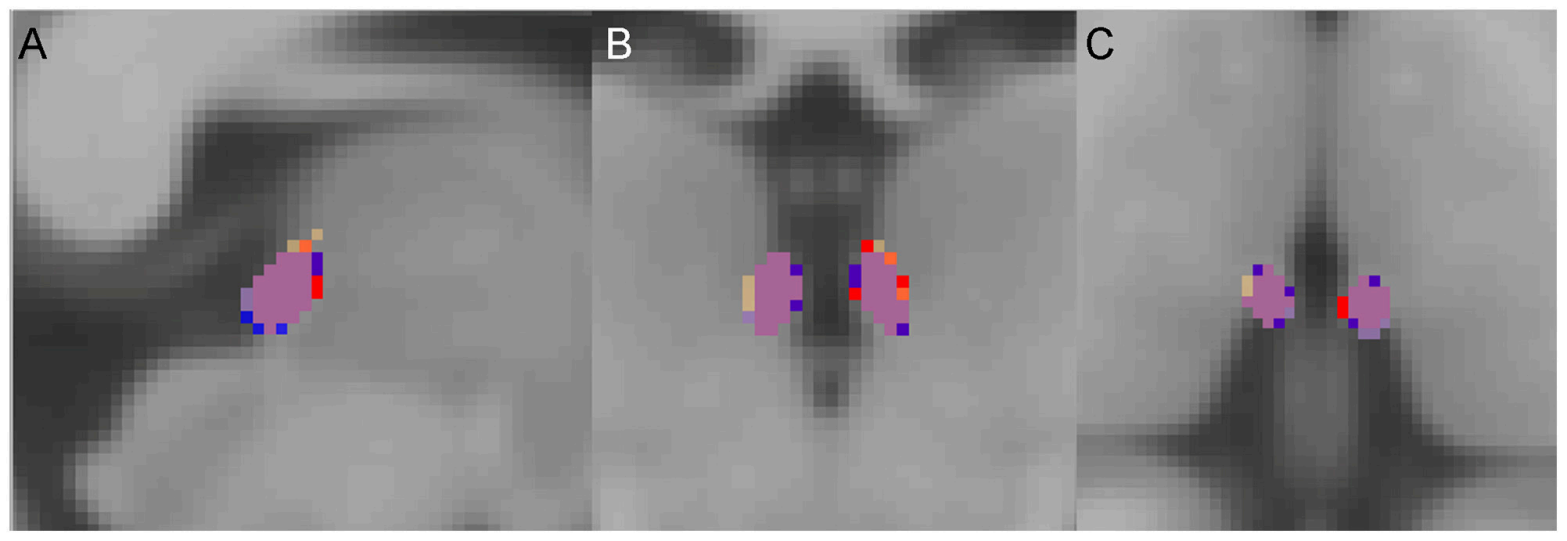
Group average habenula (Hb) probability maps. **(A)** Sagittal, **(B)** coronal, and **(C)** axial views. For each diagnostic group, the Hb probability map represents the average of the participants' unthresholded Hb maps. The overlap of the maps between the three diagnostic groups is shown in purple. For visualization a threshold of 0.1 (10%) was applied. Red = Hb probability map of the patients with schizophrenia, Orange = Hb probability map of the patients with bipolar disorder, Blue = Hb probability map of the healthy individuals.

Lastly, in order to ensure no group-level biases in the overestimation of the Hb volume along the emergence of the fasciculus retroflexus (FR) (48, 64), we computed the average probabilistic maps of the Hb, for each group, after normalizing each individual map to the MNI template (Figure 2). There was no observable overestimation along FR for any of the diagnostic groups.

### Statistical analyses

Group differences in age, sex, and IQ were examined using *t*- and χ^2^ tests as appropriate. The study sample (*n* = 140) was powered at α = 0.05 and β = 0.20 for an effect size of 0.5 for each case-control comparison. This effect size was chosen as it is large enough to be considered meaningful (65) while remaining conservative despite the reported effect sizes of approximately 0.7 in the two previous positive studies (52, 53). The normality of distribution of the continuous variables of interest was tested using one-sample Kolmogorov–Smirnov test. After confirming the normality of the Hb volume distributions within the entire sample and within each group (*p* > 0.1 for all tests), the effect of diagnosis was tested using analyses of variance models (ANOVA). We used Cohen's d to express the effect size of case-control differences. Hypotheses testing Spearman's correlations were computed between the right and left Hb volume and BPRS items of depression (item 3), suicidality (item 4), blunted affect (item 16) and emotional withdrawal (item 17); A *p*-value of 0.006 was considered as significant following Bonferroni correction for eight comparisons.

Associations between left and right Hb volume and demographic, cognitive and clinical variables [age of onset, daily antipsychotic dose (in CPZE), and lithium treatment status (binarized as on or not-on)] were assessed using Pearson's or Spearman correlation or χ^2^ tests as appropriate. Statistical significance for these latter analyses was set at *p* < 0.05 uncorrected to ensure that all potentially informative results were reported.

All analyses were conducted separately for absolute and total intracranial volume (ICV)-corrected Hb volumes. In each individual dataset, we used the FreeSurfer image analysis suite (v.5.3.0; http://surfer.nmr.mgh.harvard.edu/) to extract the ICV. Hb volumes were corrected for variation in ICV using an established formula (66): $Vol_{adj}=Vol-\;\beta^{*}\left(ICV-\;\bar{ICV}\right)$, where *Vol*_*adj*_ is the ICV-adjusted volume, *Vol* is the original uncorrected volume, β is the slope from the linear regression of *Vol* on *ICV*, and $\bar{ICV}$ is the mean ICV across all participants. As the results (i.e., group differences and correlations with clinical symptoms) were statistically unchanged, we report the findings from the analyses using absolute volumes.

## Results

Tables 1, 2 summarize the sample characteristics and Table 3 shows the mean absolute values of the Hb volumes per diagnostic group and sex. The groups did not significantly differ in age or sex (Table 1) and the patients' groups did not differ in age of onset or BPRS total scores (Table 1).

**Table 2:** Patients' clinical profile.

**BPRS items Mean (*SD*)/(range)**	**Patients with bipolar disorder *N* = 32**	**Patients with schizophrenia *N* = 68**
Somatic concern	1.62 (1.69)/(1–7)	2.04 (2.08)/(1–7)
Anxiety	2.94 (1.831)/(1–7)	2.91 (1.86)/(1–7)
Depression	2.78 (1.91)/(1–7)	2.57 (1.99)/(1–7)
Suicidality	1.72 (1.27)/(1–5)	1.62 (1.45)/(1–7)
Guilt	1.44 (0.759)/(1–3)	1.46 (1.37)/(1–7)
Hostility	1.47 (1.21)/(1–7)	1.54 (1.33)/(1–7)
Elevated mood	2.72 (2.14)/(1–7)	1.57 (1.34)/(1–7)
Grandiosity	2.42 (2.04)/(1–7)	1.84 (1.78)/(1–7)
Suspiciousness	2.59 (1.93)/(1–7)	3.29 (2.24)/((1–7)
Hallucinations	2.28 (1.61)/(1–6)	3.49 (2.28)/((1–7)
Unusual thought content	2.78 (2.18)/(1–7)	4.19 (2.19)/(1–7)
Bizarre behavior	2.50 (1.72)/(1–6)	3.26 (2.20)/((1–7)
Self- neglect	1.47 (1.10)/(1–6)	1.87 (1.47)/(1–7)
Disorientation	1.16 (0.62)/(1–4)	1.22 (0.96)/(1–5)
Conceptual disorganization	1.97 (1.53)/(1–7)	2.06 (1.49)/(1–6)
Blunted affect	1.66 (1.00)/(1–5)	2.75 (1.65)/(1–7)
Emotional withdrawal	1.19 (0.39)/(1–2)	2.26 (1.61)/(1–7)
Motor retardation	1.44 (0.80)/(1–4)	1.61 (1.21)/(1–6)
Tension	1.81 (1.42)/(1–6)	1.75 (1.38)/(1–7)
Uncooperativeness	1.22 (1.07)/(1–7)	1.38 (0.79)/(1–5)
Excitement	2.25 (2.01)/(1–7)	1.37 (1.07)/(1–7)
Distractibility	2.28 (1.74)/(1–7)	1.82 (1.26)/(1–5)
Motor hyperactivity	2.22 (1.82)/(1–7)	1.40 (1.01)/(1–6)
Mannerisms and posturing	1.55 (1.20)/(1–6)	1.65 (1.34)/(1–6)

*BPRS, Brief Psychiatric Rating Scale; SD, standard deviation; In the BPRS symptoms are rated from 1 (absent) to 7 (extremely severe)*.

**Table 3:** Absolute habenula volumes.

	**Left habenula volume**			**Right habenula volume**		
	**W**	**M**	**Total**	**W**	**M**	**Total**
Patients with schizophrenia	14.59 (4.09)	15.30 (3.28)	15.10 (3.51)	16.64 (4.02)	17.05 (3.86)	16.93 (3.92)
Patients with bipolar disorder	15.37 (4.67)	14.36 (5.14)	14.74 (4.91)	15.90 (3.62)	15.95 (4.30)	15.93 (3.99)
Healthy individuals	15.01 (3.69)	14.83 (3.95)	14.91 (3.78)	16.21 (4.24)	14.87 (3.58)	15.41 (3.86)

*Mean (Standard-deviation). W, Women; M, Men; Volumes are in mm^3^*.

### Effect of potential confounding variables

Regardless of diagnosis, the association between age and the Hb volumes was negligible and not statistically significant (all |*r*| < 0.05). Similarly, there were no differences in Hb volumes between men and women (*t* < 0.43, *p* > 0.87). Although the groups differed in IQ (Table 1), this was not associated with the Hb volumes (all |*r*| < 0.1). Analyses restricted in patients only found no significant associations between the Hb volumes and age at illness onset (all |*r*| < 0.1) or antipsychotic dose (all |*r*| < 0.15). No difference in Hb volumes was identified in patients with BD based on lithium treatment status (*t* < 0.94, *p* > 0.34, for either left or right Hb).

### Effect of diagnosis

**Figure 3:**
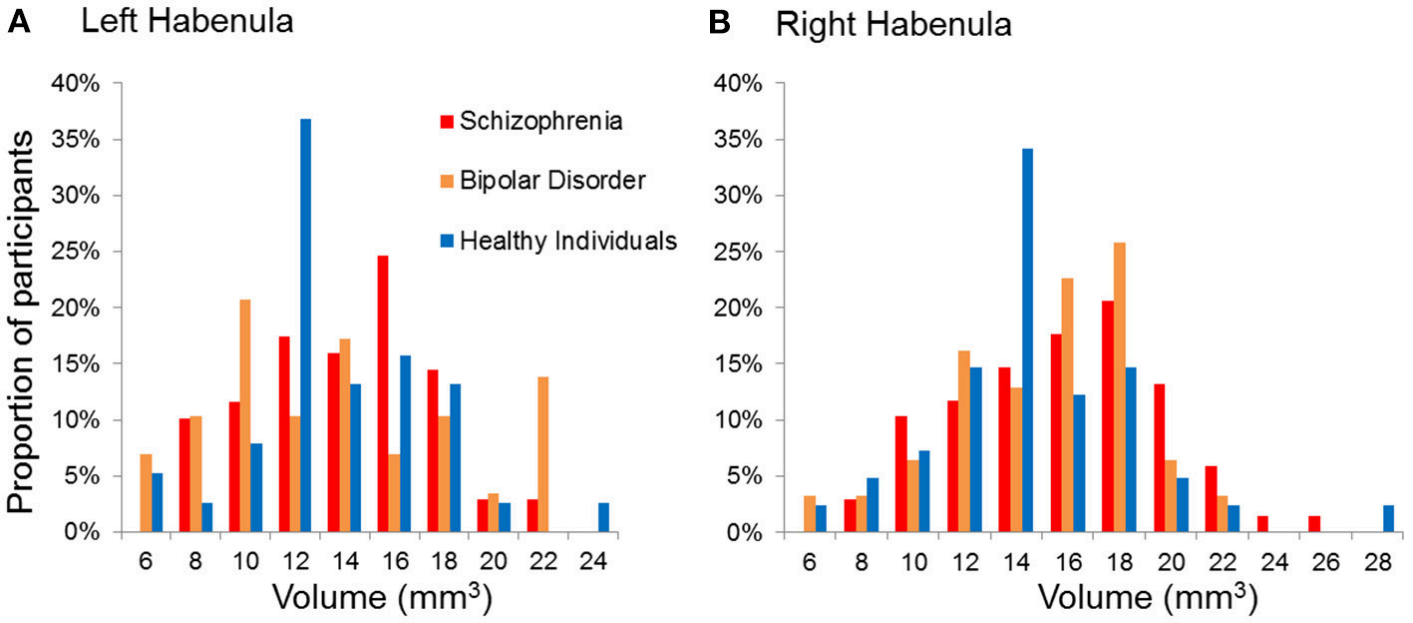
Distribution of the absolute volumes of the habenula in each diagnostic group. **(A)** Left habenula, **(B)** Right habenula.

We did not find a statistical significant effect of diagnosis in Hb volumes (Right Hb: *F* = 1.59, *p* = 0.21; Left Hb: *F* = 0.98, *p* = 0.38) (Figures 2, 3, Table 3). The results remain unchanged when we used repeated measures ANOVA with hemisphere (left, right) as the within-subject factor and diagnosis as between-subject factor. Male patients with schizophrenia had larger right Hb than healthy male participants but this analysis was only nominally significant at *p* = 0.01, uncorrected for multiple comparisons. Adding sex, age or IQ as covariates to the ANOVA models did not affect the results either. In SCZ, the effect size of case control differences was *d* = 0.14 and *d* = 0.34 for the left and right Hb volume respectively. In BD, the effect size of case control differences was *d* = −0.03 and *d* = 0.26 for the left and right Hb volume respectively.

### Correlation with clinical symptoms

**Figure 4:**
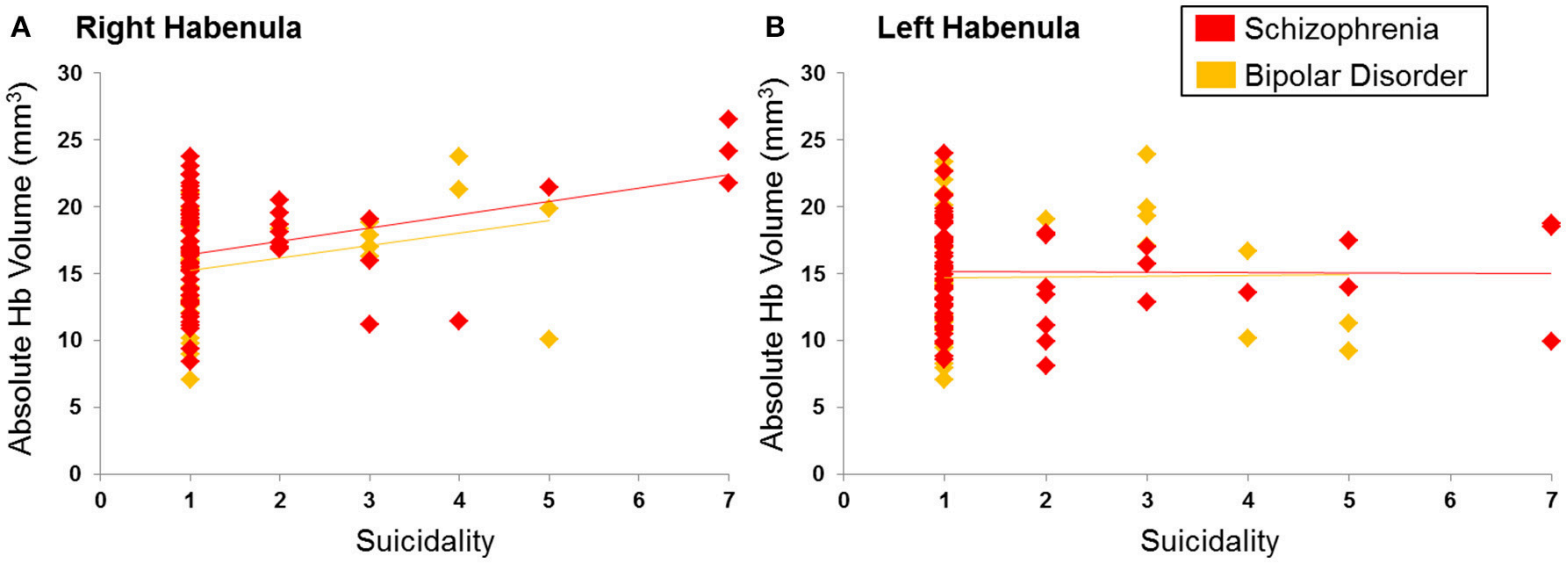
Correlation between suicidality and habenula volume. **(A)** Right habenula, **(B)** Left habenula. Suicidality was rated using the Brief Psychiatric Rating Scale from 1 indicating absence to 7 indicating severe suicidal intent and behavior. Ratings of suicidality showed a positive correlation with the right habenula volume (in mm^3^) (ρ = 0.29; *p* = 0.004) but not the left habenula volume (ρ = 0.06; *p* = 0.52).

Consistent with our hypotheses we examined correlations between the right and left Hb and BPRS items of depression, suicidality, blunted affect and emotional withdrawal. The right Hb volume was significantly and positively correlated with suicidality (Spearman's ρ = 0.29, *p* = 0.004) in the entire patient sample (Figure 4) as well as in each diagnostic group (BD: ρ = 0.34, *p* = 0.04; SCZ: Spearman's ρ = 0.25, *p* = 0.04). No association was found between the left and right Hb volume and depression, blunted affect and emotional withdrawal even at uncorrected p-values (all Spearman's |ρ| < 0.11, *p* > 0.15). The Exploratory analyses with the remaining BPRS items (Table 2) did not identify any further correlations (all |ρ| < 0.19, *p* > 0.05).

## Discussion

This is the largest neuroimaging investigation of the *in vivo* Hb morphology in BD and SCZ implemented using strict quality control criteria and an objective semi-automated segmentation method shown to yield consistent Hb boundary definitions (47). The results suggest that volumetric case-control differences are small but the variability in Hb volume may be linked to the severity of suicidality both in SCZ and BD.

To our knowledge, there are only two prior neuroimaging studies of the Hb in the clinical populations considered here. Zhang et al. (52) compared the volume, manually segmented using a geometric method, and resting state connectivity of the Hb of 15 patients with SCZ and 16 healthy individuals. They reported bilateral Hb volume reductions with large effect size (*d* = −0.84 on the left and *d* = −0.72 on the right) that did not correlate with the patients' total BPRS scores. By contrast, they found a positive correlation between overall symptom severity and the functional connectivity of the right Hb and the left mPFC. Savitz et al. (53) assessed the volume of the Hb, manually segmented based on T1w image contrast, in medicated (*n* = 15) and unmedicated (*n* = 22) patients with BD and in healthy individuals (*n* = 74). Case-control differences in Hb volume were of large effect size in unmedicated patients (*d* = −0.74 on the left and *d* = −0.66 on the right) but were negligible in medicated patients (both |*d|* < 0.1). The reason for this discrepancy remains unclear as no association was found between Hb volume and treatment variables (including lithium status). In the same study no correlation was observed between Hb volumes in patients with BD and the total scores of the Montgomery-Asberg Depression Rating Scale (67) and the Young Mania Rating Scale (68). Here, we found larger right Hb volumes in patients with SCZ or BD compared to healthy individuals; the case-control differences were of small effect size and not statistically significant. On the left, the Hb volume in SCZ was marginally larger than that of healthy individuals while case-control differences in BD were negligible. Given, the very sparse evidence-base (including the present study) the most parsimonious conclusion is that these case-control differences for the right Hb are inconsistent and probably small to moderate. The data are insufficient to allow conclusions about volumetric changes in the left Hb in SCZ and BD.

The functional significance of such Hb volume alterations is also unclear given the divergence of findings across studies; this divergence may be attributable to inter-study differences in Hb segmentation methods, in assessing the association between Hb volume and symptoms and in the variability in the clinical state of study samples. Nevertheless, the present study points to a link between the volume of the Hb and BPRS ratings of the severity of suicidal desire, intent and behaviors. Results from a recent post-mortem study of suicide victims (43) offer a speculative but heuristic interpretation of our findings. Specifically, Han et al. (43) showed downregulation of cholinergic signaling genes in the postmortem Hb tissue of 12 male Caucasian suicide victims compared to that of 11 psychiatrically healthy men. These findings are important in the context of the transdiagnostic relevance of the cholinergic system. Specifically, *in-vivo* and *ex-vivo* studies have reported persistent dysfunction involving nAChRs) in MDD (69) while similar abnormalities in BD have been observed during depressive episodes only (70). Hypofunction of nAChRs has also been consistently reported in SCZ (71, 72) where it has been associated with cognitive and hedonic impairment (73, 74). Han et al. (43) implicate the cholinergic MHb-IPN pathway, which is phylogenetically conserved (31, 32, 75) and has been shown to regulate response to acute and chronic stress across species (40). Acetylcholine (ACh) is synthesized by choline acetyltransferase (CHAT) and its release in the MHb-IPN pathway is mediated by presynaptic nAChRs). Depression models of chronic restraint or learned helplessness, lead to decrease CHAT levels and its corresponding gene expression in the Hb of rats while CHAT knockout mice show anhedonia-like behaviors (43). Although previous research has mainly focused on the role of LHb in positive valence processing (34, 39) our results indicate a potential significant contribution from the MHb particularly for suicidality.

There are several limitations to this study. The spatial pattern of myelin content of the Hb is higher toward the stria medialis and lower in the ventromedial portions (64). Because of this pattern, any segmentation that is based primarily on anatomical image contrast may underestimate the voxels located at the ventral medial portion of the Hb. This observation is likely to account for the lower *in-vivo* Hb volumes reported here and in previous studies (46, 47, 50–53) compared to *ex-vivo* estimates (31, 46, 49). The volumetric measurements obtained here did not distinguish between the lateral and medial subdivision of the Hb because such distinction was beyond the resolution of our imaging approach. Image contrasts between Hb sub-regions have been shown two *ex-vivo* MRI studies (46, 76). A single high-resolution *in-vivo* 7T anatomical MRI study has reported that it is possible to differentiate between the LHb and MHb (77) but this distinction was not based on clear boundary definitions nor was it validated against histology. Differentiating subdivisions of the Hb using MRI *in-vivo* is still an unmet need.

The majority of the patients in our study were medicated. We did not find an association between medication type and dose and Hb volumes in either patient group. This is in line with all available neuroimaging findings with the exception of a single study in which Hb volume reduction was observed in unmedicated but not in medicated patients with BD (53). Nevertheless the possibility that medication exposure may have minimized differences between diagnostic groups cannot be fully refuted. The cross-sectional nature of this study does not allow inferences as to whether possible Hb volume reductions are vulnerability traits or whether they arise after illness onset. Future directions include confirmation of these findings in larger samples using longitudinal designs and highlight the importance of testing for alterations in functional engagement and connectivity of the Hb in SCZ and BD.

## Data availability statement

The raw data supporting the conclusions of this manuscript will be made available by the authors, without undue reservation, to any qualified researcher.

## Author contributions

SF, GD, and JX contributed to the conception and design of the study. MS, GD, JJ, and J-WK performed the analyses. MS wrote the first draft of the manuscript. MS, SF, and GD wrote sections of the manuscript. All authors contributed to manuscript revision, read and approved the submitted version.

### Conflict of interest statement

The authors declare that the research was conducted in the absence of any commercial or financial relationships that could be construed as a potential conflict of interest.
